# Heterogeneous Skin Phantoms for Experimental Validation of Microwave-Based Diagnostic Tools

**DOI:** 10.3390/s22051955

**Published:** 2022-03-02

**Authors:** Jasmine Boparai, Milica Popović

**Affiliations:** Department of Electrical and Computer Engineering, McGill University, Montreal, QC H3A 0E9, Canada; jasmine.boparai@mail.mcgill.ca

**Keywords:** biological tissues, dielectric properties, dielectric measurement, liposarcoma, microwaves, nonsyndromic basal cell carcinoma (BCC), tissue-mimicking phantoms, tumor

## Abstract

Considerable exploration has been done in recent years to exploit the reported inherent dielectric contrast between healthy and malignant tissues for a range of medical applications. In particular, microwave technologies have been investigated towards new diagnostic medical tools. To assess the performance and detection capabilities of such systems, tissue-mimicking phantoms are designed for controlled laboratory experiments. We here report phantoms developed to dielectrically represent malign skin lesions such as liposarcoma and nonsyndromic multiple basal cell carcinoma. Further, in order to provide a range of anatomically realistic scenarios, and provide meaningful comparison between different phantoms, cancer-mimicking lesions are inserted into two different types of skin phantoms with varying tumor–skin geometries. These configurations were measured with a microwave dielectric probe (0.5–26.5 GHz), yielding insight into factors that could affect the performance of diagnostic and detection tools.

## 1. Introduction

Over the past decade, microwave reflectometry techniques have been researched for diagnosis and early-stage characterization of malignancies such as subcutaneous masses, skin burn injuries and cancerous lesions in the brain, breast and skin [1,2,3,4,5]. In particular, we focus on techniques that exploit the reported inherent dielectric contrast of healthy and malignant tissues in the microwave frequency range [6,7] to identify cancerous lesions or anomalies. Low-power microwave-based techniques have the advantages of being safe, cost effective and portable. The current modalities which are considered as gold standards, such as magnetic resonance imaging (MRI), X-rays, computed tomography methods or CT scanning and ultrasound, each have their shortcomings. For example: X-rays and CT scans involve ionizing radiation, limiting frequent screening; MRI is expensive and not suitable for frequent mass screenings; ultrasound imaging is operator-dependent and requires real-time interpretation [8,9,10,11,12]. Further, the nonspecificity of available techniques for skin cancer requires biopsies, which are uncomfortable to the patient and invasive. The goal of microwave-based diagnosis is to provide additional insight into the nature of the lesion under investigation, thereby reducing the number of needed biopsies [13]. The development of microwave-based systems for the accurate characterization of abnormalities can assist the physician in diagnostics by providing additional information that can facilitate decision-making with improved confidence. Consequently, the possibly cancerous anomalies can be identified at their early stage, increasing the success rate of the subsequent treatment.

Successful adoption of microwave diagnostic systems requires their systematic testing and validation in a controlled laboratory environment. Here, well-designed tissue phantoms play an important role in the preclinical trial stage. Based on different material compositions, fabrication processes, complexity, stability and cost, several tissue-mimicking phantoms have been reported. These phantoms vary considerably in their shape and structure, ranging from simple geometries such as homogeneous models to heterogeneous models with realistic shapes. In Ref. [14], the authors realized several human tissues with acetonitrile mixtures over the wide frequency range from 0.5 to 18 GHz. In the study of [15], Triton X-100 and distilled water solutions were investigated over a wide-band frequency to reproduce the dielectric properties of different types of breast tissues. In addition to being subject to dehydration, the liquid-based phantoms pose a challenge for inhomogeneous structure construction [16]. Garrett and Fear proposed carbon and rubber mixtures-based phantoms, which exhibit a wide range of dielectric properties and hence mimic a variety of tissues up to 10 GHz [17,18]. Although these phantoms are electrically and mechanically stable, their material composition is expensive and the fabrication process is complex [19]. In addition, semisolid phantoms are widely adopted for emulating various tissues like fat, muscle and skin due to their ability to achieve better approximations of the targeted tissues. The heterogeneous and stable breast phantom composed of multiple tissues such as skin, fat, muscle and spherical inclusion was reported in [20]. Jelly-type or semisolid-type materials mimicking the dielectric properties of human skin tissues are also commercially available [21]. These are stable over a longer period of time. Recently, there has been an increasing interest in easily reproducible 3D-printed phantoms; however, often the materials that are used in their fabrication are not dielectrically characterized [22]. Previously, we constructed and characterized tumors in skin with diameter sizes ranging from 2 mm to 10 mm at 2 mm increments with underlying thick and thin skin along with an irregular-shaped tumor [23]. 

Our present study is motivated by the need for stable, anatomically and dielectrically accurate heterogeneous phantoms that emulate rare and realistic configurations. This will contribute to the improvement of the efficacy of the emerging diagnostic devices in screening such rare cases, along with the frequently occurring skin cancer forms. Thus, in this work, we present a methodology of constructing phantoms imitating realistic scenarios such as liposarcoma and nonsyndromic multiple basal cell carcinoma [24,25,26,27]. Liposarcoma is a rare type of cancer which sometimes develops as a subcutaneous mass in the fat layer just below the skin. It can begin anywhere in the body but is most commonly found in the abdomen, thigh and behind the knee. Additionally, in some rare conditions like nonsyndromic multiple basal cell carcinoma, an individual can develop multiple lesions at once. Often, surgical excision is advised to identify the nature of the tumor or diagnose the lesion with the help of X-rays, MRI, CT scan or ultrasound. In certain cases, there is a risk associated with surgical removal, for example, ruptured lesions can leave cells behind in the tissue which can be carried to other parts of the body through the bloodstream. Microwave techniques based on nonionizing radiation, and low in power, hold promise as diagnostic aids which could help the dermatologist in the decision-making process and in the detection of the subcutaneous lesions. In order to test the capability of microwave diagnostic tools in distinguishing lesions from healthy skin, we constructed phantoms by incorporating tumors in two different types of skin: oil–gelatin (fabricated in-house) and Probingon AB (commercially available). Each phantom model was tested with two tumor sizes: 10 mm and 2 mm in diameter, and 2 mm in thickness for both cases.

## 2. Materials and Methods

### 2.1. Modeling of Phantoms

**Figure 1:**
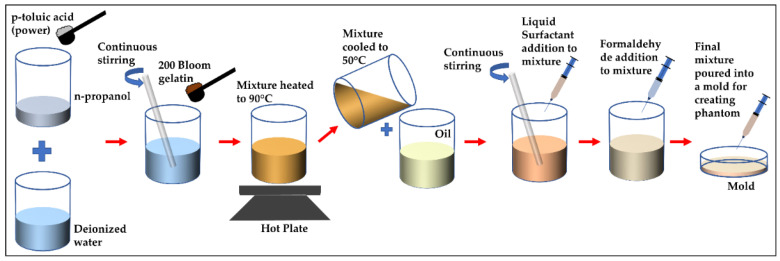
Illustration of fabrication procedure for proposed oil–gelatin phantoms.

For our study, we emulated rare conditions like liposarcoma and multiple basal cell carcinoma with oil-in-gelatin-based tissue-mimicking materials that have the ability to accurately emulate the dispersive dielectric properties (the frequency dependence of parameter values) of human tissues such as skin, fat and tumor. Moreover, with these materials, we realized heterogeneous configurations to construct realistic anatomical structures to be characterized over the wide frequency range. The oil–gelatin phantoms were fabricated according to the guidelines published in [28]. For completeness, the fabrication procedure is illustrated in Figure 1 and the corresponding steps are briefly listed in Table 1.

To represent the liposarcoma condition, the phantoms were constructed in three stages. In the first stage, we created a fat layer by pouring the fat-mimicking material into the cylindrical mold and a coin-shaped (small, shallow cylinder) void was left at the top of the fat surface, later to be filled up with the tumor-mimicking material. Before proceeding to the next stage, we left the fat to congeal for 24 h. In the second stage, the void was filled with the tumor-like material and allowed to congeal overnight. In the final stage, skin-like material was added on the top of the fat-layer-containing tumor. The thickness of the subcutaneous fat and skin layer varies depending upon number of factors such as body location, age and gender [29,30]. For this case, we selected the thickness of fat and skin to be 7 mm and 1 mm, respectively, and the diameter of 38.5 mm, which is the diameter of container mold. The two tumor sizes considered for comparison were 10 mm and 2 mm in diameter, each with a 2 mm thickness. 

**Figure 2:**
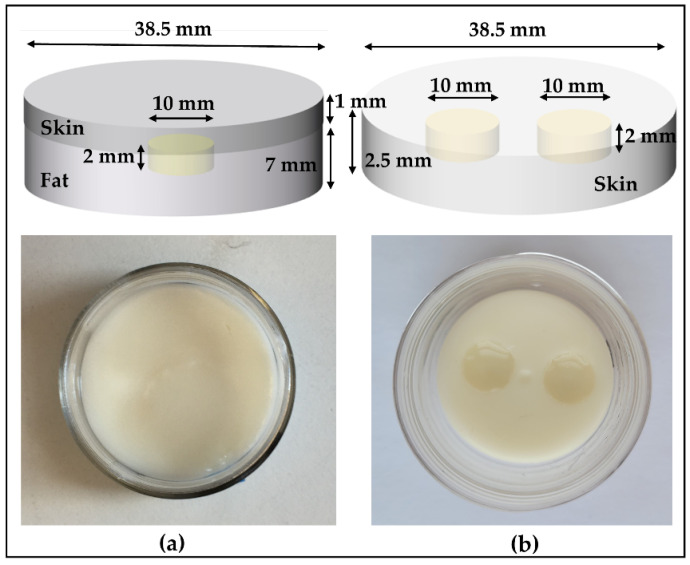
Oil–gelatin phantoms, with sketches shown on the top and the top-view photograph on the bottom: (**a**) Tumor (10 mm) embedded in fat underneath the skin and (**b**) multiple tumors in skin.

For simulating the condition with multiple BCC lesions, we followed similar steps as mentioned above. In the first stage, the skin-like material is poured into the container with two coin-shaped voids left at the top surface of the skin, which are later filled with the tumor-mimicking material. In this case, the skin thickness is 2.5 mm and diameter of the entire testing sample is 38.5 mm. Both tumors have a thickness of 2 mm and a diameter of 10 mm. The sketch and the fabricated phantoms depicting both conditions are shown in Figure 2. These phantoms have a shelf-life of several weeks when plastic-wrapped or placed in an airtight container. The phantoms are fabricated according to the compositions in Table 2 [31].

In an additional experiment, we incorporated tumors in two different skin-mimicking materials: oil–gelatin (following the recipe above) and the Probingon AB [32]. The latter is a commercially available skin phantom with a jelly-like consistency, allowing us to easily incorporate the tumors, and has been characterized for skin-like dielectric properties in the microwave range. We considered three different tumor–skin arrangements: tumor with a top surface coplanar with the skin, tumor within the skin and tumor raised slightly above the plane of the skin surface. The thickness and diameter of each skin phantom are 2.5 mm and 38.5 mm, respectively. Each tumor phantom was 2 mm thick with two diameter sizes: 10 mm and 2 mm. To avoid dehydration, the phantoms were plastic-wrapped, placed in air-tight containers and stored in the refrigerator.

**Figure 3:**
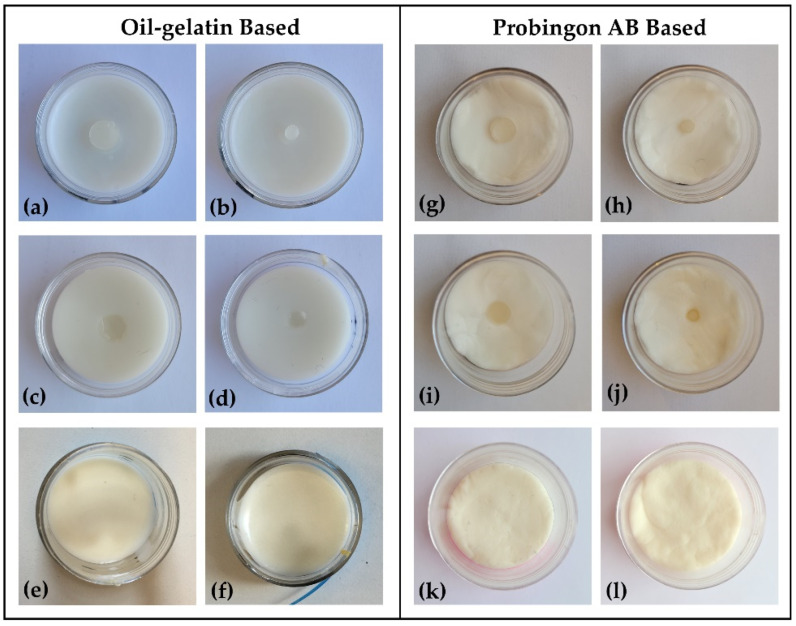
Photograph (top view) of oil–gelatin tumor phantoms in (**a**–**f**) oil–gelatin and (**g**–**l**) Probingon AB skin. Two tumor sizes in three arrangements are shown: 10 mm diameter (**a**,**g**) raised out of skin; (**c**,**i**) aligned with the skin surface; (**e**,**k**) embedded within the skin; with the same arrangements are shown for the smaller 2 mm tumor in figures (**b**,**h**); (**d**,**j**) and (**f**,**l**), respectively.

Figure 3 shows the photograph of both types of skin (oil–gelatin and Probingon AB) with oil–gelatin tumors raised beyond, aligned with the skin surface and embedded within the skin. 

### 2.2. Characterization Methodology

**Figure 4:**
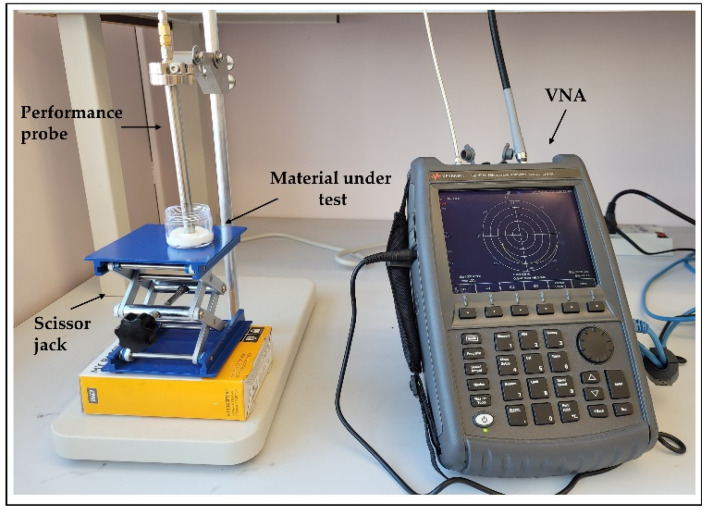
Dielectric measurement setup showing the open-ended performance coaxial probe (left) and a FieldFox vector network analyzer; material under test (MUT) is placed on scissor jack.

The dielectric properties of the proposed tissue-mimicking phantoms were measured using an open-ended coaxial probe [33]. The measurement system (Figure 4) consists of a performance probe (Keysight Technologies, model N1501A) suitable for semisolid materials and a vector network analyzer (VNA) (Keysight Technologies, model N9918A, commonly known as FieldFox Handheld Microwave Analyzer [34]). The open-ended performance probe is connected to the port of the VNA with a coaxial cable which is flexed and stabilized before calibration and measurements. The probe is locked in the mounting bracket of the probe stand to minimize the movement-induced reading errors. A sample elevator stage was used to move the sample under test towards the probe. The dielectric measurements were carried out over the entire VNA frequency range, 500 MHz–26.5 GHz, and over 1001 frequency points. The reflection coefficient (S11) obtained from the VNA are converted to real ($\varepsilon_{r}^{\prime }$) and imaginary part ($\varepsilon_{r}^{\prime \prime }$) of complex permittivity ($\varepsilon_{r}$) using Keysight’s materials measurement N1500A software suite. All measurements were performed at room temperature (23 °C).

A standard three-load calibration using air, short (metal block in Keysight probe kit) and load (deionized water) was used before conducting the measurement of the dielectric properties. The calibration was validated by measuring the dielectric properties of known materials (deionized water and air) before and after each measurement. Calibration was refreshed between the repeated measurements to increase the measurement accuracy, and air was used as the refreshing standard. The power level used was −10 dBm.

Uncertainty analysis was done at each frequency by computing the repeatability and accuracy, as reported in [35,36]. Error due to drift and cable movement is not included in the measurements since our setup is fixed. Repeatability was calculated as the standard deviation of mean of 10 repeated measurements on deionized water and averaging the values over the complete frequency range. Accuracy is calculated as the average percentage difference between the measured values and the reference models reported in the literature over the entire frequency range from 500 MHz to 26.5 GHz. In our study, we tested the accuracy of the measurements using deionized water, as it has well-known dielectric properties presented in the literature [37]. Repeatability uncertainty for permittivity and conductivity was calculated as 0.26% and 0.45%, respectively. Accuracy uncertainty was determined to be 1.78% for permittivity and 1.47% for conductivity. This resulted in the total combined uncertainty of 1.8% and 1.5% for permittivity and conductivity, respectively. 

## 3. Results and Discussion

In this section, we have assessed the dielectric measurement results of the proposed tissue-mimicking phantoms and validated them using reference models obtained from the literature. Since the dielectric properties are dependent on frequency and temperature, we monitored the temperature of the calibration and validation liquid, i.e., deionized water and the temperature of the sample under test (22.7 ± 0.4 °C), thus ensuring consistent measurements temperature-wise.The complex permittivity ($\varepsilon_{r}$), which is comprised of real ($\varepsilon_{r}^{\prime }$) and imaginary ($\varepsilon_{r}^{\prime \prime }$) parts representing the relative permittivity and loss factor, respectively, of the realized phantom models, was computed at room temperature following the guidelines of MINDER [38]. 

The conductivity ($\sigma_{s}$) is related to the loss factor ($\varepsilon_{r}^{\prime \prime }$) and computed using Equation (1) as

(1) $$\sigma_{s}=2\pi f\varepsilon_{r}^{\prime \prime }\varepsilon_{0}$$

where *f* is the frequency of the operation in hertz and $\varepsilon_{0}$ (8.854 × 10^−12^ farad/meter) is the permittivity of free space. The dielectric properties of the reference malignant BCC [39] and fat [40] tissues were obtained using the one pole Cole–Cole model described in Equation (2) and the Cole–Cole parameters given in Table 3.

(2) $$\varepsilon_{r}=\varepsilon_{r}^{\prime }-j\varepsilon_{r}^{\prime \prime }=\varepsilon_{\infty }+\frac{\Delta \varepsilon }{1+{\left(j\omega \tau \right)}^{1-\alpha }}+\frac{\sigma_{s}}{j\omega \varepsilon_{0}}$$

where ω = 2πf is the angular frequency in radians per second, $\varepsilon_{\infty }$ is permittivity of skin at optical frequencies, Δε is magnitude of skin dielectric dispersion, τ (ps) is the relaxation time, α is the measure of broadening dispersion and $\sigma_{s}\;\left(S/m\right)$ is the skin conductivity. The Cole–Cole parameters ($\varepsilon_{\infty }$, ∆ε, τ, α, $\sigma_{s}$) are determined by minimizing the function given as:

(3) $$c=\frac{\Sigma_{i=1}^{N}\;\left|\frac{\varepsilon_{r}^{\prime }\left(\omega_{i}\right)-\varepsilon_{rc}^{\prime }\left(\omega_{i}\right)}{\left(\varepsilon_{r}^{\prime }\left(\omega_{i}\right)\right)}\right|+\Sigma_{i=1}^{N}\;\left|\frac{\varepsilon_{r}^{\prime \prime }\left(\omega_{i}\right)-\varepsilon_{rc}^{\prime \prime }\left(\omega_{i}\right)}{\left(\varepsilon_{r}^{\prime \prime }\left(\omega_{i}\right)\right)}\right|}{N}$$

where N is the number of frequency points, $\varepsilon_{r}^{\prime }\left(\omega_{i}\right)$ and $\varepsilon_{r}^{\prime \prime }\left(\omega_{i}\right)$ are values measured at frequency $\left(\omega_{i}\right)$ and the values of $\varepsilon_{rc}^{\prime }\left(\omega_{i}\right)$ and $\varepsilon_{rc}^{\prime \prime }\left(\omega_{i}\right)$ are obtained from (2). The fitting procedure is performed in MATLAB using the Levenberg–Marquardt algorithm [39]. 

The resulting data, shown in the sections that follow, are plotted with mean and standard deviations in measurements across the complete microwave frequency range of interest. We performed 10 consecutive measurements on each MUT by placing the probe at the same point and mean, and two standard deviations are calculated. The mean values are represented by lines and these lines are bordered by ± standard deviations (95.5% confidence interval) represented by a shaded area. Before and after the MUT dielectric measurement, the procedure was recalibrated by measuring the dielectric properties of known materials (air and deionized water). We divided our measurement study in three parts, as detailed in the following subsections.

### 3.1. Study 1: Tumors Embedded within the Fat–Skin Layer—Liposarcoma

**Figure 5:**
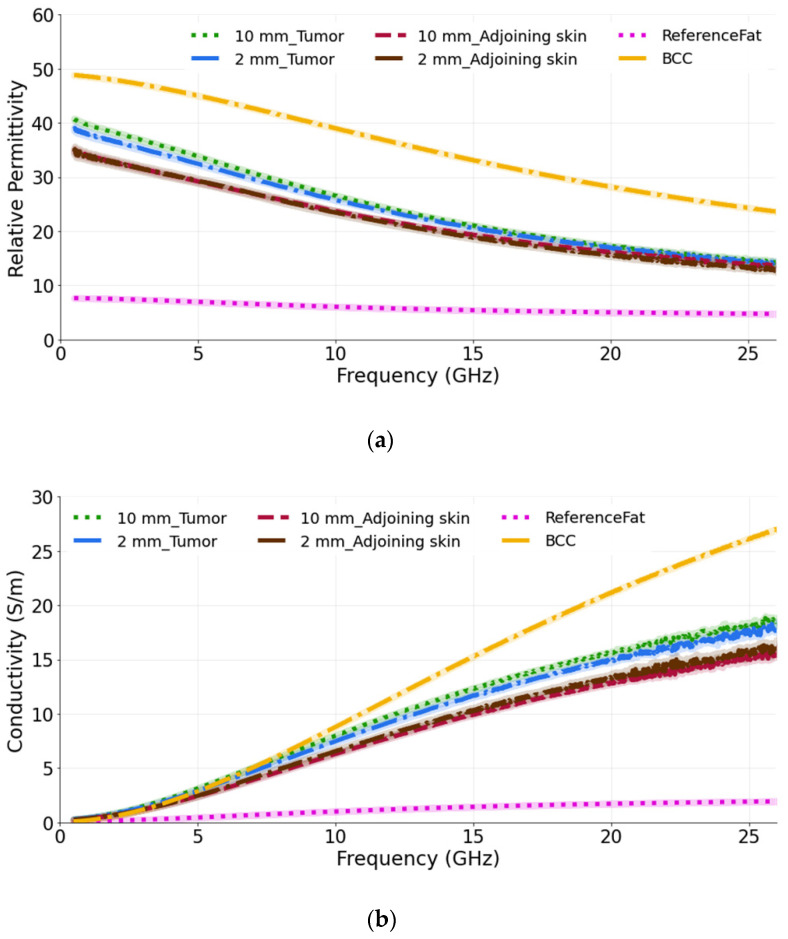
(**a**) Relative permittivity and (**b**) conductivity measurements of tumors present in the fat underneath the skin and adjoining skin compared with the reference BCC and fat data from [39,40]. For each phantom model, two tumor sizes are taken: 10 mm and 2 mm.

In our first study, we measured phantom models emulating the liposarcoma condition. This study was done to analyze the dielectric response when the lesion is in the fat layer underneath the skin. Two separate models were taken: one with a 10 mm tumor (diameter) and the other with a 2 mm tumor (diameter) to observe the ability of the probe to sense large and small tumors. The thickness of both tumors is 2 mm. The probe was held lightly against the skin under which the tumor was present, and it was ensured that the entire aperture of the probe was in contact with the skin. The relative permittivity and conductivity are plotted in Figure 5a,b. From the graphs, it is observed that the measured dielectric values are nearly the same for the two tumor sizes. The measurements were also performed on the adjoining skin of each tumor and it can be seen that there is a slight difference in the dielectric properties of the adjoining skin and the tumor phantoms up to a frequency of 15 GHz in terms of permittivity, which can be utilized to identify the lesion. This difference shows that the underlying tumor and fat layers influence, as expected, the result, as the probe is then averaging (with unknown weights) the dielectric properties of the skin, fat and tumor. Similarly, for the conductivity, the measured value remains approximately the same for both tumor sizes over the entire frequency band. However, when the adjoining skin is measured, the difference is observed between the tumor in the fat under the skin and in the adjoining skin from 10 GHz to 26.5 GHz. 

The computed results are also compared with the reference BCC and reference fat values obtained from the literature [39,40]. As expected, the measured permittivity and conductivity values are lower than the reference BCC values and higher than the reference fat tissue, as the probe perceives a value averaged among the individual components of the complex dielectric distribution. 

### 3.2. Study 2: Multiple-Lesion Arrangement on the Skin Surface

**Figure 6:**
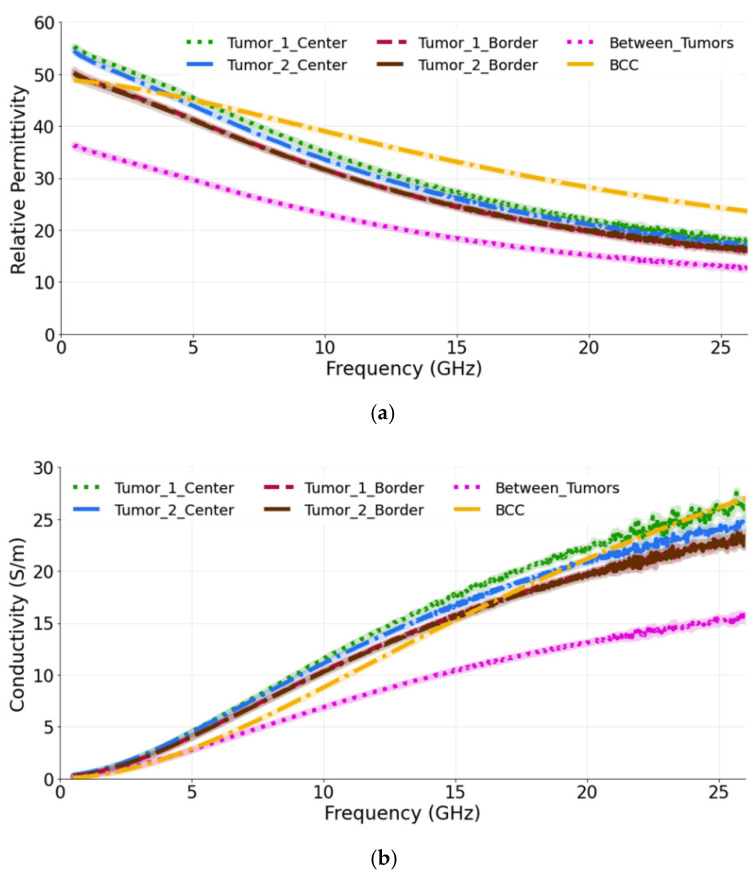
(**a**) Relative permittivity and (**b**) conductivity measurements at the center of each tumor, at the border of each tumor and in-between the tumors compared with the reference data from [39]. The diameter of both tumors is 10 mm.

The aim of second study is to observe how the presence of more than one tumor affects the dielectric measurements. Therefore, in this case more than one tumor (10 mm in diameter) is placed so that its upper surface is coplanar with the surface of the skin. We label them as T1 and T2. The measurements were conducted for three probe locations: at the center, at the border of each tumor and in-between (equidistantly) the two tumors. As is seen from the Figure 6a,b, when the dielectric measurements are performed at the center of each tumor, the relative permittivity and conductivity are higher in comparison to when the measurements are conducted at the border of each tumor. Again, these results are expected, since when measuring at the border, the probe averages the dielectric properties of both skin and tumor, as both of these materials are present within its sensing volume. Similar, but not identical, averaging of properties occurs when the probe is placed between the tumors, thereby sensing yet another heterogeneous dielectric distribution. We observe that the permittivity of tumors, when measured at the center, closely matches the reference BCC value. The percentage difference between T1 measured at the center and the reference BCC is calculated as 14.6% and 32.7% for the permittivity and conductivity, respectively. Similarly, T1, when measured at the border, has the difference of 20.9% and 22.9% with reference BCC for the permittivity and conductivity, respectively. The percentage difference measured in-between T1 and T2 and the reference BCC is 41.2% and 29.6% for the permittivity and conductivity, respectively.

### 3.3. Study 3: Testing Two Skin Phantoms: Oil–Gelatin and Probingon AB

The objective of this study is to identify the detection capability of the probe when the tumor is placed in two different types of skin (oil–gelatin and Probingon AB) at three different locations relative (in alignment with the surface of skin, embedded in the skin and raised out of skin) to the skin. For each case, we have considered tumor sizes of 10 mm and 2 mm in diameter. 

#### 3.3.1. Case 1: Tumors Aligned with the Skin Surface

**Figure 7:**
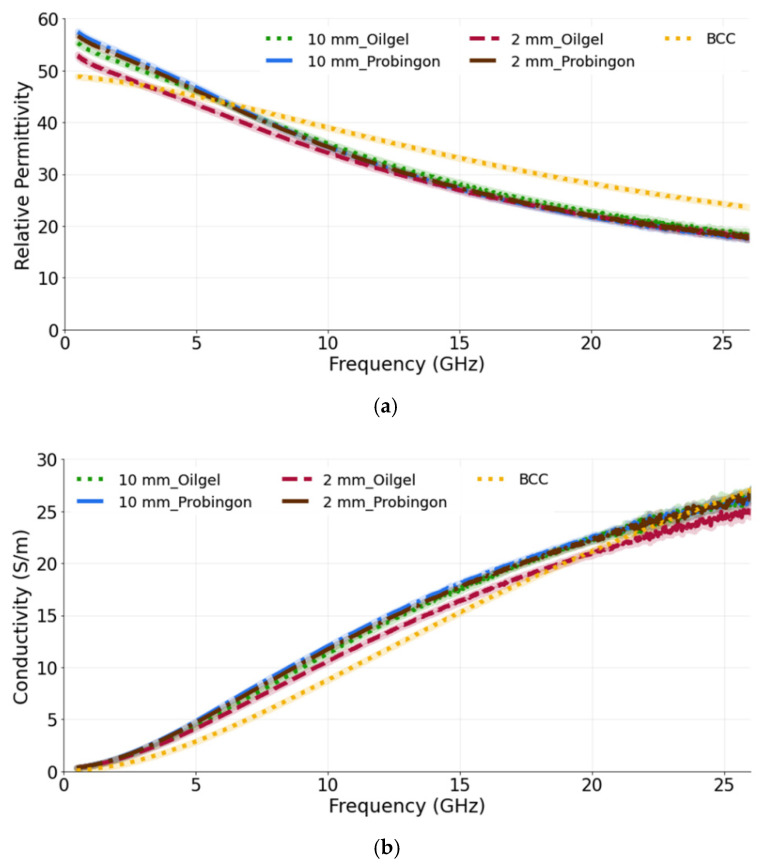
(**a**) Relative permittivity and (**b**) conductivity measurements of tumors present in two types of skin: oil–gelatin and Probingon AB. The results are compared with the reference data from [39]. For each case, measurements are performed with two tumor sizes (10 mm and 2 mm in diameter) and the tumor is in alignment with the top surface of the skin.

Figure 7a,b show the relative permittivity and conductivity, respectively, for tumors when placed in alignment with the skin. It can be seen that, for both tumor sizes and their placement in two different types of skin, the dielectric properties are approximately the same. This demonstrates the ability of the probe to identify the tumor (10 mm or 2 mm) regardless of the skin phantom used. While comparing the measured results with the reference BCC dielectric properties, we can see that both models exhibit a similar trend. The computed percentage difference between the 10 mm tumor in alignment with the oil–gelatin skin and the reference BCC is 12.6% and 31.2%; for the 10 mm tumor aligned with the Probingon AB skin model, the values are 15.7% and 37.2% for the permittivity and conductivity, respectively.

#### 3.3.2. Case 2: Tumor Embedded within the Skin

**Figure 8:**
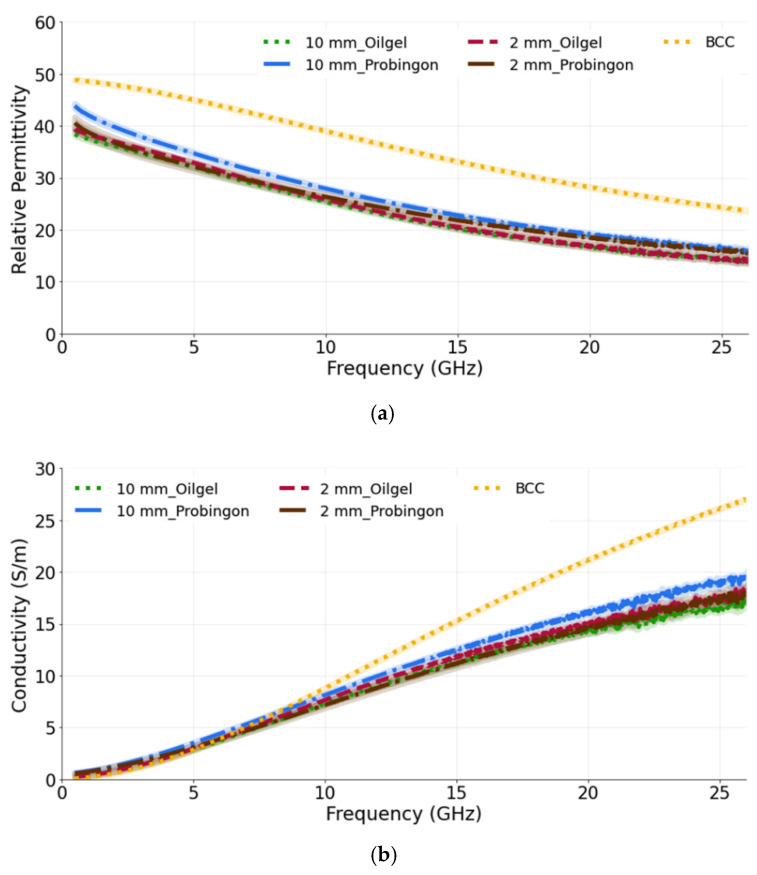
(**a**) Relative permittivity and (**b**) conductivity measurements of tumors present in two types of skin: oil–gelatin and Probingon AB. The results are compared with the reference data from [39]. For each case, measurements are performed with two tumor sizes (10 mm and 2 mm in diameter) and the tumor is embedded within the skin.

In the next case, the tumors (10 mm and 2 mm in diameter) are placed within the oil–gelatin-based and Probingon AB skins. The measured relative permittivity and conductivity are given in Figure 8a,b, both showing values that are lower than the reference BCC tumor values. Clearly, the skin layer surrounding the tumor is sensed by the probe and also contributes to the overall result. The percentage difference for the 10 mm tumor embedded in the oil–gelatin skin, the 10 mm tumor embedded in the Probingon skin and the reference BCC is 35.8% and 26.3%, and 28.2% and 37.0%, in terms of the permittivity and conductivity, respectively. 

#### 3.3.3. Case 3: Tumor Raised out of the Skin

**Figure 9:**
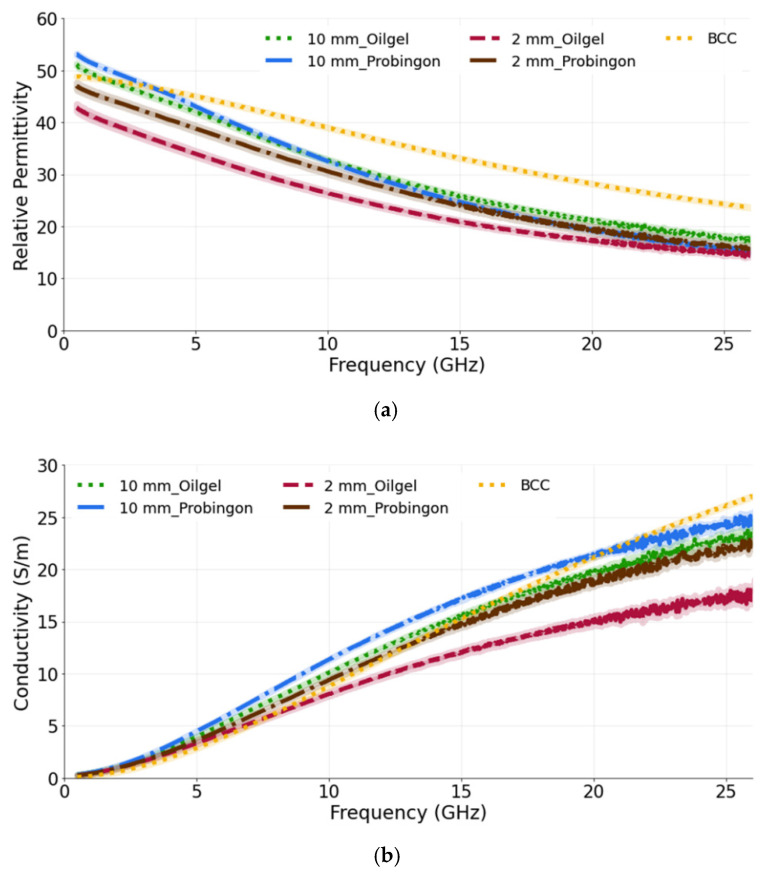
(**a**) Relative permittivity and (**b**) conductivity measurements of tumors present in two types of skin: oil–gelatin and Probingon AB. The results are compared with the reference data from [39]. For each case, measurements are performed with two tumor sizes (10 mm and 2 mm in diameter) and the tumor is raised out of skin.

For the last case, the tumor is raised out of skin, again considering two skin phantoms and the 10 mm and 2 mm diameter tumor models. We observe that the 10 mm tumors in both skin models exhibit the same dielectric permittivity and conductivity (as shown in Figure 9a,b). The 10 mm tumor in the Probingon AB and oil–gelatin has perceived higher dielectric properties and are more closely matched to the reference BCC than the 2 mm tumor. In the case of the smaller tumor, the probe’s sensing volume clearly includes more of the skin material. The percentage difference between the 10 mm tumor raised out of the skin models and the reference BCC are 17.8% and 24.1% (oil–gelatin) and 20.9% and 30.7% (Probingon AB) for the permittivity and conductivity, respectively. 

## 4. Conclusions

Tissue-mimicking phantoms are needed for the validation and assessment of new diagnostic prototypes in controlled laboratory environments and prior to clinical trials. In this study, we developed and examined the dielectric performance of realistic skin tumor phantom models aimed at mimicking these tissues for applications in the microwave frequency range 0.5–26.5 GHz. The phantoms simulated different conditions: liposarcoma condition, where we placed the tumor in the fat underneath the skin, and nonsyndromic multiple basal cell carcinoma condition, where more than one tumor was placed in alignment with the skin surface. Further, we investigated the use of different skin-mimicking materials by placing oil–gelatin-based tumors in two skin models (oil–gelatin and Probingon AB). Each phantom model was tested with two tumor sizes (10 mm and 2 mm in diameter) in order to assess the probe’s ability to identify the tumor, as its size will impact the complex dielectric distribution present in the probe’s sensing volume. 

Thus, the goal of our study was to characterize stable heterogeneous phantom models which dielectrically and anatomically represent several skin and tumor geometries. The results have an impact on the meaningful interpretation of the test results for the microwave diagnostic tools aimed to assist the dermatologist in the decision-making process. The oil–gelatin phantoms were fabricated with off-the-shelf components and compared to the commercially available Probingon AB skin phantom. The characterization was performed over the 0.5–26.5 GHz range. The resulting phantom model data were compared with the reference excised tissues from the literature, the dispersive properties of which were evaluated using one-pole Cole–Cole parameters. Encouragingly for the microwave-based diagnostic tools under development, our results indicate that, even when the skin layers surrounding the tumor result in heterogeneous dielectric distribution within the probe’s sensing volume, the microwave probe is still able to identify the tumor lesions.

## Figures and Tables

**Table 1:** A summary of steps for constructing oil-in-gelatin-based phantoms.

Step 1.	Add p-toluic acid to n-propanol and heat the solution.
Step 2.	Mix solution of p-toluic acid and n-propanol to deionized water at room temperature.
Step 3.	Add gelatin to the obtained mixture and heat the mixture at 90 °C until it becomes transparent.
Step 4.	Cool the mixture in water bath to 50 °C.
Step 5.	Mix oil (50% safflower and 50% kerosene) separately and heat up to 50 °C.
Step 6.	Combine mixtures of step 4 and 5.
Step 7.	Add Ultra Ivory and formaldehyde to above mixture.
Step 8.	Pour the resultant mixture into mold and allow it to solidify.

**Table 2:** Composition used in the fabrication of phantoms shown in Figure 2 [31].

Target Tissue	p-Toluic Acid (g)	n-Propanol (mL)	Deionized Water (mL)	200 Bloom Gelatin (g)	Formadehyde (37% by Weight) (g)	Oil (mL)	Ultra Ivory Detergent (mL)
Fat	0.133	6.96	132.7	24.32	1.53	265.6	12.0
Skin	0.294	28.69	279.5	50.02	3.33	98.6	5.86
Tumor	0.346	17.0	328.0	58.67	3.72	38.4	2.00

**Table 3:** One pole Cole–Cole parameters to compute dielectric data of malignant BCC and fat from the literature.

Tissue Type	$\varepsilon_{\infty }$	Δε	τ (ps)	$\sigma_{s}\;\left(S/m\right)$	α
Malignant BCC [39]	6	43.04	7.66	0.05	0.08
Fat Group 3 [40]	4.031	3.645	14.12	0.083	0.055
